# Effect of Puumala hantavirus infection on human umbilical vein endothelial cell hemostatic function: platelet interactions, increased tissue factor expression and fibrinolysis regulator release

**DOI:** 10.3389/fmicb.2015.00220

**Published:** 2015-03-24

**Authors:** Marco Goeijenbier, Joost C. M. Meijers, Fatih Anfasa, Jeroen M. Roose, Cornelia A. M. van de Weg, Kamran Bakhtiari, Heikki Henttonen, Antti Vaheri, Albert D. M. E. Osterhaus, Eric C. M. van Gorp, Byron E. E. Martina

**Affiliations:** ^1^Department of Viroscience, Erasmus MC, RotterdamNetherlands; ^2^Department of Experimental Vascular Medicine, Academic Medical Center, University of AmsterdamAmsterdam, Netherlands; ^3^Department of Plasma Proteins, Sanquin Research, AmsterdamNetherlands; ^4^Department of Internal Medicine, Faculty of Medicine, Universitas IndonesiaJakarta, Indonesia; ^5^Artemis One Health Institute, UtrechtNetherlands; ^6^Metla, Finnish Forest Research Institute, VantaaFinland; ^7^Department of Virology, Haartman Institute, University Of HelsinkiHelsinki, Finland

**Keywords:** hantavirus, hemostasis, platelets, endothelial cells, thrombin generation, hemorrhagic fever with renal syndrome, HFRS

## Abstract

Puumala virus (PUUV) infection causes over 5000 cases of hemorrhagic fever in Europe annually and can influence the hemostatic balance extensively. Infection might lead to hemorrhage, while a recent study showed an increased risk of myocardial infarction during or shortly after PUUV infection. The mechanism by which this hantavirus influences the coagulation system remains unknown. Therefore we aimed to elucidate mechanisms explaining alterations seen in primary and secondary hemostasis during PUUV infection. By using low passage PUUV isolates to infect primary human umbilical vein endothelial cells (HUVECs) we were able to show alterations in the regulation of primary- and secondary hemostasis and in the release of fibrinolysis regulators. Our main finding was an activation of secondary hemostasis due to increased tissue factor (TF) expression leading to increased thrombin generation in a functional assay. Furthermore, we showed that during infection platelets adhered to HUVEC and subsequently specifically to PUUV virus particles. Infection of HUVEC with PUUV did not result in increased von Willebrand factor while they produced more plasminogen activator inhibitor type-1 (PAI-1) compared to controls. The PAI-1 produced in this model formed complexes with vitronectin. This is the first report that reveals a potential mechanism behind the pro-coagulant changes in PUUV patients, which could be the result of increased thrombin generation due to an increased TF expression on endothelial cells during infection. Furthermore, we provide insight into the contribution of endothelial cell responses regarding hemostasis in PUUV pathogenesis.

## Introduction

Puumala virus (PUUV), a hantavirus carried by chronically infected bank voles, is the causative agent of an estimated 5000 cases yearly of viral hemorrhagic fever in Europe (Vapalahti et al., 2003; Vaheri et al., 2013a). Hantaviruses are rodent-borne, negative stranded, RNA viruses belonging to the *Bunyaviridae* family, which may cause two types of disease in humans (Goeijenbier et al., 2013). In Europe and Asia, hantavirus infection causes hemorrhagic fever with renal syndrome (HFRS), characterized by renal failure and bleeding complications. In North and South America, hantavirus infection causes the hantavirus cardiopulmonary syndrome (HCPS) where patients present with severe acute respiratory distress (Sargianou et al., 2012). Changing ecological factors determine fluctuations in hantavirus epidemiology resulting in sudden increases in incidence, for instance through increased food availability, prolonged virus survival and decreased biodiversity (Reusken and Heyman, 2013). Recent epidemiological studies reported an overall incidence increase of PUUV infections in Europe (Heyman and Vaheri, 2008).

Although PUUV infections have a low case fatality rate (<1%) and in literature the virus is often described as the least virulent of the pathogenic viruses within the hantavirus genus, PUUV infections can cause severe disease in healthy adults, which may require a long recovery period lasting up to 1 year (Schmaljohn and Hjelle, 1997). Furthermore, several reports described cases with severe (hemorrhagic) complications like pituitary gland hemorrhage, hematemesis and gastro-intestinal bleedings (Eckerle et al., 2012; Antonen et al., 2013). In contrast to these bleeding complications, a recent study from Sweden reported increased risk for acute myocardial infarction shortly after PUUV infection (Connolly-Andersen et al., 2014). Given the high incidence in Northern Europe, acute myocardial infarction as a complication of PUUV infection could have a major impact in endemic areas. In light of both bleeding and thrombotic events that might complicate PUUV infections, we hypothesized that endothelial cells, also the target cells for hantaviruses and the major regulators of coagulation and inflammation, play a central role in the pathogenesis of the disease (Zhou et al., 2003).

During hantavirus infection drastic alterations in the coagulation system have been observed (Laine et al., 2014). Clinical studies focusing on primary and secondary hemostasis during hantavirus disease showed thrombocytopenia in both HFRS and HCPS, a decreased plasma activity of coagulation factors II, V, VIII, IX, and X in acute HFRS patients, prolongation of the prothrombin and activated partial thromboplastin time, increased thrombin generation and D-dimer levels and a decrease in ADAMTS13 activity in acute PUUV patients (Lee, 1987; Mackow and Gavrilovskaya, 2009; Laine et al., 2011; Mustonen et al., 2013). The ability to infect endothelial cells by hantaviruses has been demonstrated both *in vitro* and *in vivo* (Yanagihara and Silverman, 1990; Pensiero et al., 1992; Toro et al., 1998). Although infection does not lead to cytopathic changes, several studies observed endothelial cell dysfunction during hantavirus infection (Yanagihara and Silverman, 1990; Pensiero et al., 1992; Mackow and Gavrilovskaya, 2009), ranging from increased clinical markers of a stressed endothelium *in vivo* (sICAM-1, VWF and circulating endothelial cells; Han et al., 2010; Krautkramer et al., 2014), to increased permeability and decreased HUVEC integrin ligand migration *in vitro* (Gavrilovskaya et al., 2002; Geimonen et al., 2002; Taylor et al., 2013).

Integrin ανβ3, experimentally proven to be the receptor for hantavirus infection, is abundantly present on the surface of endothelial cells (Gavrilovskaya et al., 1999; Song et al., 2005). Infection with pathogenic hantaviruses is suggested to result in the loss of function of the ανβ3 integrin (Wang et al., 2012), but also an increased ανβ3 expression on cultured endothelial cells and platelets has been observed (Liu et al., 2008). Furthermore Gavrilovskaya et al. (2010) studied the adherence of quiescent platelets to Sin Nombre and Hantaan virus infected endothelial cells seems to be the result of virus binding to the ανβ3 integrin present on platelets (Gavrilovskaya et al., 2010).

How the abnormalities in the primary (thrombocytopenia) and secondary hemostasis (increase in thrombin generation and raised D-Dimer levels) are induced in PUUV infected patients and the mechanism by which Old-World hantaviruses cause hemorrhage and/or renal failure remain largely elusive (Vaheri et al., 2013b). Lack of specific treatment and an effective vaccine makes understanding of the pathophysiology of hantavirus infection an important medical need, especially with the recently discovered association of PUUV with cardiovascular disease (Connolly-Andersen et al., 2014). Therefore, we have used an integrated approach to study changes in primary and secondary hemostasis using an *in vitro* endothelial cell model.

## Materials and Methods

### Cells

VeroE6 cells (American Type Culture Collection, USA) were grown in Dulbecco’s Modified Eagle Medium (DMEM) containing 10% fetal bovine serum (FBS, Lonza, the Netherlands), 100 U/ml penicillin-streptomycin solution, 1% Hepes buffer and 1% sodium bicarbonate (all from Gibco, Life Sciences, USA). Human umbilical vein endothelial cells (HUVECs) were harvested from umbilical veins, which were kindly provided by Erasmus MC birth center. Briefly, umbilical cords were stored in sterile 500 ml PBS supplemented with gentamycin (50 μg/ml; Leo Pharmaceutical, Denmark). Veins were rinsed with PBS containing 50 U/ml heparin (Leo Pharmaceutical). Subsequently, cells were detached with 0.1% collagenase solution (C6885, Sigma Aldrich, USA). Cell suspension was collected in a sterile 50 ml tube followed by two times centrifugation (5 min 300 *g*). The cell pellet was re-suspended in HUVEC medium (human endothelial-SFM medium; Invitrogen, Life Sciences, USA) containing 10% human serum (Lonza), 20% filtrated FBS (Lonza); penicilin/streptomycin 100 U/ml, 20 ng/ml fibroblast growth factor (Peprotech, USA) and 10 ng/ml of endothelial cell growth factor (Peprotech). HUVEC cell suspensions were cultured in flasks pre-coated with 20 μg/ml of fibronectin (Roche, the Netherlands). Only cells up to passage four, from one specific donor, were used for this study. Identity of the endothelial cells was confirmed by flow cytometry using *Ulex europeus* lectin, anti-CD31 and Von Willebrand Factor (VWF) staining and immunoblot.

### Anti-Sera

We made use of the following antibodies and conjugates: polyclonal rabbit anti- VWF, HRP labeled polyclonal goat anti-rabbit IgG and polyclonal rabbit anti-mouse (All from Dako, the Netherlands). FITC labeled monoclonal anti-CD31 (Sigma Aldrich, USA), polyclonal rabbit anti-CD41 (Perbio Science, the Netherlands), polyclonal rabbit anti-CD3 (Dako), polyclonal rabbit anti-PUUV nucleoprotein (BEI Resources, USA), monoclonal anti-PUUV glycoprotein (HY Test, Finland), monoclonal anti-ανβ3 integrin (Abcam, UK), monoclonal anti-vitronectin (Novus Bio, USA), polyclonal rabbit-anti PAI-1 (Bio Connect, the Netherlands), polyclonal rabbit anti-tissue factor (TF; Bio Connect), human serum from a recovered PUUV case described in Goeijenbier et al. (2011) retrieved after informed consent and ethical board approval. Antibodies and conjugate were diluted in dilution buffer, which consisted of PBS with 0.5% bovine serum albumin, 2% NaCl and 1% normal goat serum.

### Virus Infection

#### Virus Isolation

Lungs of *Myodes glareolus* from Konnevesi, Finland, infected with PUUV were homogenized in DMEM (10% w/v) and 100 μl was added onto a 70–80% monolayer of VeroE6 cells and incubated for 60 min at 37^∘^C in 5% CO_2_. The supernatant was discarded and cells were washed three times and incubated with fresh veroE6 medium for an additional 5 days. Virus stocks up to passage four were created by centrifugation (10 min 400 *g*) of the supernatant to create a cell free virus stock. Virus titer was determined using immune peroxidase reaction (IPOX) and TCID_50_ was calculated using the Karber formula (Kärber, 1931). Infectious virus was inactivated using beta-propiolactone (BPL; Sigma Aldrich, USA; 1:4000 v/v) at 4^∘^C for 24 h. Subsequently, BPL was inactivated for 1 h at 37^∘^C. All virus stocks were stored at -80^∘^ until use. All experiments were conducted under biosafety instructions required regarding work with live PUUV. Vesicular stomatitis virus (VSV) strain Indiana, propagated also on VeroE6 cells, was kindly provided by Dr. Bart Haagmans (Erasmus MC).

#### Infection Kinetics and Dynamics

Human umbilical vein endothelial cell were seeded into 24-well- (2.4 × 10^5^ cells) or 96-well plates (4 × 10^4^ cells; Corning, USA) depending on the experiment. Confluent monolayers were infected with a multiplicity of infection (MOI) of 0.5 or 3, with infectious and inactivated (BPL-inactivated) virus or a normal medium control for 60 min at 37^∘^C in 5% CO_2_. After incubation, the supernatant was discarded and cells were washed three times with RPMI 1640 (Gibco, Life Sciences). Fresh medium was added as described earlier. For VWF and plasminogen activator inhibitor type-1 (PAI-1) quantification, medium did not contain FCS but was supplemented with 4% sterile filtered bovine serum albumin (BSA, Sigma Aldrich, USA) to avoid addition of fetal calf VWF and PAI-1 (Zoellner et al., 1996). To quantify the percentage of infected cells we used an in house developed IPOX procedure. HUVEC were washed three times with PBS. Cells were fixed with absolute -20^∘^C methanol and incubated at -20^∘^C for 30 min. After fixation, methanol was discarded and cells were incubated for 30 min at 37^∘^C with 100 μl of 0.05% H_2_O_2_ in PBS, to block endogenous peroxidases. Subsequently, cells were washed three times with PBS and incubated for 60 min with polyclonal rabbit anti-PUUV nucleoprotein antibody (1:500). Cells were washed with PBS 0.05% tween followed by incubation with HRP-labeled goat anti-rabbit IgG conjugate (1:500). Color development was achieved by addition of 3-amino-9-ethylcarbazole (AEC) substrate (AEC dissolved in dimethylformamide buffered with acetate buffer of pH 5). Percentage of infected cells was determined by manual counting.

For the quantification of viral replication we used a standard line of in house generated PUUV RNA run-off transcripts, as described for West-Nile virus (Lim et al., 2013). Briefly, RNA run-off transcripts were generated using a segment amplified with pan-hantavirus degenerative PCR primers from (Johansson et al., 2010). PCR products were separated on 1% agarose gel and bands of correct size were collected for DNA gel extraction using the MinElute Gel Extraction Kit Protocol (Qiagen, USA). DNA fragments were cloned into the pCR4 vector using the TOPO^®^ TA Cloning KIT (Life Technologies) and One Shot^®^ TOP10 chemically competent *Escherichia coli* were transformed with the recombinant vector (QIAGEN) according to manufacturer’s protocol. At least five colonies were collected for further analyses. Plasmid DNA was purified using MinElute DNA purification kit (QIAGEN). Plasmid DNA was linearized by restriction digestion (NotI for the negative strand RNA and PstI for the positive strand RNA). Run-off transcripts (*in vitro* transcripts) were synthesized using T3 RNA polymerase for negative strand and T7 RNA polymerase for positive strand (MEGAscript^®^ T3 and T7 transcription kits, Life technologies), followed by DNase treatment (Ambion^®^ TURBO DNA-free^TM^ Life Technologies), according to manufacturer’s manual. The amount of RNA in the stock was determined using NanoDrop^®^ and serially diluted. Copy numbers in the standards were calculated using RNA concentration and sequence length with help of an online calculator ().

### Platelet Binding Assay

#### Platelet Collection

To study interaction between PUUV and platelets, platelets were collected according to the protocol described in Gavrilovskaya et al. (2010) with minor modifications. Briefly, blood was collected in 0.105 M (end concentration) sodium citrate tubes (BD-plymouth, UK) supplemented with 1 μM prostaglandin E1 (Cayman Chemical, USA) to block platelet activation. Platelet-rich plasma (PRP) was prepared by centrifugation for 15 min at 700 ×*g* at 25^∘^C. Subsequently, platelets were pelleted for 15 min at 1300 × *g* at 25^∘^C. Platelets were washed twice and resuspended with modified hepes buffer (25 mM Hepes, 137 mM NaCl, 0.1% Albumin and 1 μM prostaglandin E1 pH 7.4) and counted by a hematocytometer.

#### Platelet HUVEC Binding

Platelets (10^8^ per ml) were incubated with infected HUVEC (96 wells plate; PUUV, BPL inactivated PUUV or mock control) for 30 min at 37^∘^C. After incubation, monolayers were washed three times with RPMI and cells were fixed with formalin. After fixation, formalin was discarded and cells were incubated for 30 min at 37^∘^C/5% CO_2_ with PBS 0.05% H_2_O_2_. After three washing steps cells were incubated with rabbit polyclonal anti-human CD41a antibody (1:500). The following steps were as described earlier for IPOX. After incubation with HRP-labeled goat anti-rabbit conjugate (1:1000) TMB was added to the wells for substrate reaction. After 10 min reaction was stopped by addition of 0.5 M sulphuric acid and optical density (OD) was measured at 450 nm using Tecan ELISA reader. CD41a expression OD was calculated by subtracting the blanc OD value (wells incubated without platelets but with detection antibody and conjugate). Rabbit polyclonal anti-CD3 (1:500) served as an isotype control.

#### Platelet PUUV Binding

To test if changes in CD41a expression was related to direct binding between PUUV and platelets, a mechanism shown in Hantaan and Andes virus infection (Gavrilovskaya et al., 2010), a pull down assay was designed. To this end, we first coated ELISA plates with PUUV or a control virus (VSV; 100 μl of 10^6^ virus particles in DMEM at 4^∘^C overnight) followed by platelet incubation (10^7^ platelets). Subsequently, cells were washed five times with PBS and the bound platelets were quantified by using a platelet detection antibody (anti-CD41a 1:500 in dilution buffer) followed by a conjugate substrate reaction. CD41a expression was calculated by subtraction of the OD measured in the wells without platelet incubations (blanco) to correct for direct (a-specific) anti-CD41a antibody binding to PUUV and anti-CD3 was used as isotype control. Subsequently, ELISA plates were coated with mouse monoclonal anti-PUUV glycoprotein- or isotype control antibody (IgG2 corona virus; 1:500 in PBS at 4^∘^C overnight) followed by incubation with PUUV to capture the virus followed by platelet incubation, detection antibody and conjugate substrate reaction. Thirdly, to further confirm platelet PUUV binding, ELISA plates were coated with an anti-platelet antibody (1:500 in PBS at 4^∘^C overnight), followed by incubation with fresh isolated platelets and eventually an incubation step with PUUV or VSV followed by a hantavirus detection antibody (mouse mAB anti-PUUV-glycoprotein 1:500). After washing substrate reaction was achieved by conjugate addition and TMB reaction steps. As a final step we studied the potential blocking of platelet binding by PUUV particles by the addition of a blocking step with polyclonal human anti PUUV serum. PUUV coated plates and plates coated with 5 days old virus free VeroE6 medium were incubated with a polyclonal PUUV serum (1:50) from a case described in Goeijenbier et al. (2011) or with a PUUV IgG negative control human serum from a healthy volunteer (also 1:50). The following platelet binding steps and CD41 detection were the same as in the earlier experiments.

### Von Willebrand Factor and Plasminogen Activator Inhibitor Type-1 Quantification

After infection, in a 24-well plate, supernatants (500 μl) were subsequently removed and cells were lysed, after three washing steps, using a 15 min incubation with 500 μl PBS 1% TritonX-100 followed by centrifugation (10 min 400 ×*g*). Cell-free supernatants and supernatant from cell lysates were measured using a PAI-1 antigen and VWF ELISA kits according the manufacturer’s instructions (both from Zymugen, Hyphen Biomed, France).

### Tissue Factor Expression and Activity

#### Tissue Factor Cell Surface Expression

Human umbilical vein endothelial cell in 96 wells plates were fixed with 4% formalin and incubated with rabbit polyclonal anti-TF antibody (1:500) followed by incubation with the respective conjugate (1:500). After washing TMB was added for substrate reaction and reaction was stopped after 10 min by addition of 0.5 M of sulphuric acid. OD 450 nm value was measured on Tecan ELISA reader.

#### Tissue Factor Cell Lysate Concentration

Cell lysates were prepared as described in Section “Von Willebrand Factor and Plasminogen Activator Inhibitor Type-1 Quantification.” ELISA plates were coated with a mixture of 50 μl cell lysate and 50 μl PBS over night at 4^∘^C together with a standard curve of recombinant TF (Innovin; Siemens Healthcare Diagnostics, Germany). After blocking wells were incubated with rabbit polyclonal anti-TF antibody (1:500) followed by incubation with the respective conjugate (1:500). After washing TMB was added for substrate reaction and reaction was stopped after 10 min by addition of 0.5 M of sulphuric acid. OD 450 nm value was measured on Tecan ELISA reader.

#### Thrombin Generation

Thrombin generation in platelet-poor plasma (PPP) was measured directly on HUVEC surface, in a 96 well plate, by recalcification of 80 μl of pooled citrated plasma from healthy donors added to the monolayer of infected and uninfected cells. In summary, cells were washed three times with RPMI and 80 μl freshly thawed plasma was added to the monolayer together with 60 μl of HEPES buffer (25 mM Hepes, 137 mM NaCl, 0.1% albumin). On the same plate a serial dilution of recombinant TF (Innovin; Siemens Healthcare Diagnostics, Germany) in the absence of cells. Finally, 60 μl of HEPES calcium [25 mM Hepes, 137 mM NaCl, 0.1% Albumin, 38 mM CaCl(2)] was added to plasma. Directly after recalcification, OD 450 nm value was measured using a Tecan ELISA reader in a kinetic cycle measuring every 45 s for 1 h. Thrombin generation time was defined as the time at half-maximal OD.

### Vitronectin – PAI-1 Complex Levels

ELISA plates were coated with anti-vitronectin antibody (1:500 in PBS at 4^∘^C overnight), incubated with supernatant from PUUV infected or non-infected HUVEC followed by incubation with polyclonal anti-PAI-1 antibody (1:500) and subsequent conjugate-substrate reaction. PBS incubation was used as a blanc control and anti-CD3 antibody (1:500) incubation as an isotype control.

### Statistics

All statistical analyses were performed using GraphPad Prism 5.01 for Windows. When comparing two groups we made use of a Student’s *t*-test or Mann–Whitney *U*, depending on the distribution of the data. For the comparison between multiple groups non-parametric Kruskal-Wallis test was used with Dunn’s multiple comparison test or a one-way ANOVA with Tukey’s multiple comparisons test, depending on the distribution of the data.*P*≤ 0.05 were considered significant.

## Results

### PUUV Infects and Replicates in Primary Endothelial Cells

**FIGURE 1:**
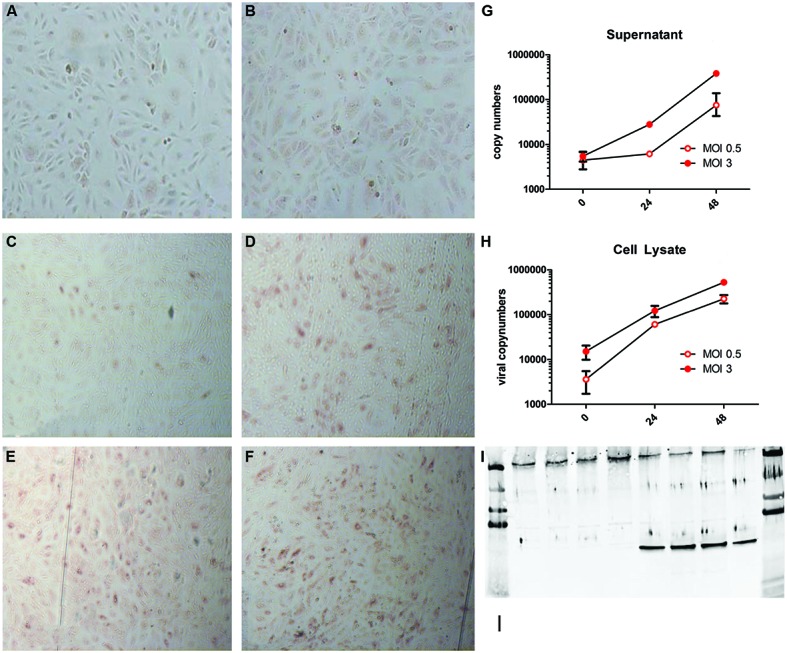
**Puumala virus (PUUV) immune peroxidase (IPOX) staining and viral copy numbers after infection with a multiplicity of infection (MOI) of 0.5 and 3.** Mock infected cells **(A,B)** showed no red peroxidase staining for PUUV nucleoprotein after 24 **(A)** and 48 **(B)** hours. Twenty-four hours after infection with MOI 0.5 **(C)** a small number of cells stained positive, which, increased at 48 h **(D)**. Infection with MOI 3 resulted in more infected cells after 24 h **(E)**, which increased slightly at 48 h post infection **(F)**. Analysis of viral replication showed a more than 2-log increase of viral copy numbers in both supernatant **(G)** and cell lysates **(H)**, suggesting active viral replication. Bars represent SE of the mean. BPL inactivation of the virus lead to no increase in viral copy numbers in the supernatant and a negative IPOX staining (data not shown). Furthermore infection was confirmed by western blot for the presence of the PUUV nucleoprotein in the cell lysate and Von Willebrand Factor (VWF) to confirm the character of the endothelial cells **(I)**. The first four lanes show control wells with only one band present at the upper side of the blot (VWF). The last for lanes show the presence of both VWF and the PUUV nucleoprotein (∼55 kDa 10ug/lane). Data are representative of three independent experiments.

To prevent PUUV from *in vitro* loss of virulence, virus stocks of not more than four passages were prepared. Freshly isolated HUVEC were infected with MOI 0.5 and 3. The PUUV infected and replicated in HUVEC, as is summarized in **Figure 1**. Non-infected cells (**Figures 1A,B**) showed no red peroxidase staining, confirming specificity of the PUUV-staining. From 24 h post infection with a low MOI infection (0.5) onward (**Figure 1C**) only a small percentage (±10%) of the cells were infected, which strongly increased after 48 h (**Figure 1D**), resulting in 50% of infected stained cells. Twenty-four hours after infection at a MOI of 3 about 40–50% of cells were infected (**Figure 1E**), which increased further to 80% by 48 h (**Figure 1F**). Comparable kinetics were seen when viral RNA copy numbers were determined. To this end, viral RNA numbers were estimated both in supernatant (**Figure 1G**) and cell lysate (**Figure 1H**). At both MOIs the number of viral RNA increased significantly (2-Log) after 48 h (Kruskal-Wallis; *p* = 0.0028), in the supernatant as well as in the cell lysate, confirming active viral replication. Viral replication reached a plateau at 72 h post infection.

Furthermore western-blot analysis of the cell lysate for PUUV nucleoprotein confirmed infection of HUVEC (**Figure 1**). The viral copy numbers in the supernatant or cell lysate of HUVEC incubated with BPL inactivated virus served as a control for non-replicating virus. Consistently, the RNA copy numbers did not increase over time indicating the efficient inactivation of the virus by BPL treatment. Efficient inactivation was confirmed by negative IPOX staining of the HUVEC incubated with BPL inactivated PUUV (data not shown).

### Increased CD41a Expression after Incubation of Platelets on HUVEC upon PUUV Infection

**FIGURE 2:**
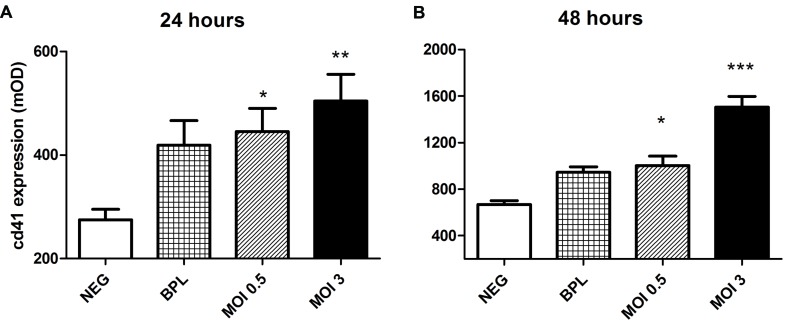
**Increased platelet binding to PUUV infected HUVEC.** Increased optical density (OD) of CD41a was measured both 24 **(A)** and 48 **(B)** hours after infection with both low and moderate MOI and platelet incubation on HUVEC surface. *p* values (*p < 0.05, **p < 0.01, ***p ≤ 0.001) are the result of one way ANOVA testing with Tuckey’s multiple comparison posttest. Bars represent the SE of the mean. Incubation with an isotype control antibody (polyclonal anti-CD3) did not lead to increased OD on infected or control HUVEC. Data are representative of three independent experiments.

Gavrilovskaya et al. (2010) reported the potential of Hantaan and Andes virus to bind quiescent platelets via ανβ3 integrin. Since this observation is of much importance in further understanding the alterations in primary hemostasis and its role in disease mechanisms in HFRS we decided to confirm this mechanism for PUUV using a different approach. First we assessed the ability of quiescent platelets to bind to PUUV infected HUVEC (**Figure 2**). Binding of platelets was determined by measuring the intensity of CD41a (platelet glycoprotein IIb), a heterodimeric integral membrane protein present only on platelets and megakaryocytes. CD41a expression was significantly higher on the HUVEC monolayer after infection with a MOI of 0.5, or 3 compared to the control. Detection of CD41a expression did not differ when platelets were not added to the HUVEC monolayers, suggesting that there was no non-specific anti-CD41a binding to infected cells. Furthermore there was no difference in OD values when an isotype control (anti-CD3) was used to detect platelets. Based on CD41a expression, statistically significant differences were measured between infected wells and wells incubated with virus free VeroE6 medium (negative control) at 24 h post infection (one way ANOVA, MOI 3 vs. NEG *p* < 0.01, MOI 0.5 vs. Neg *p* < 0.05). After 48 h of infection this difference in CD41a expression was also significant between MOI 3 infected wells and the BPL inactivated virus control (one way ANOVA, MOI 3 vs. NEG *p* < 0.001 and MOI 3 vs. BPL *p* < 0.001; MOI 0.5 vs. NEG *p* < 0.05). Taken together, the data indicate that platelets bind to cultures incubated with PUUV. HUVEC incubated with BPL did show a trend to increased platelet CD41a expression (**Figure 2**), but this was not statistically significant.

### Von Willebrand Factor (VWF) is not Increased During PUUV Infection of HUVEC

We measured VWF antigen in cell free supernatant and VWF expression on the surface of infected HUVEC. Increased VWF production may be a general inflammatory response of endothelial cells that could be evoked as a result of PUUV infection. However, at the time points where platelet binding increased, HUVECs infected with PUUV showed no alteration in VWF activity, as determined by ELISA, in neither the supernatant nor the cell lysate (data not shown) compared to BPL or negative control.

### Platelets Bind Directly to PUUV

**FIGURE 3:**
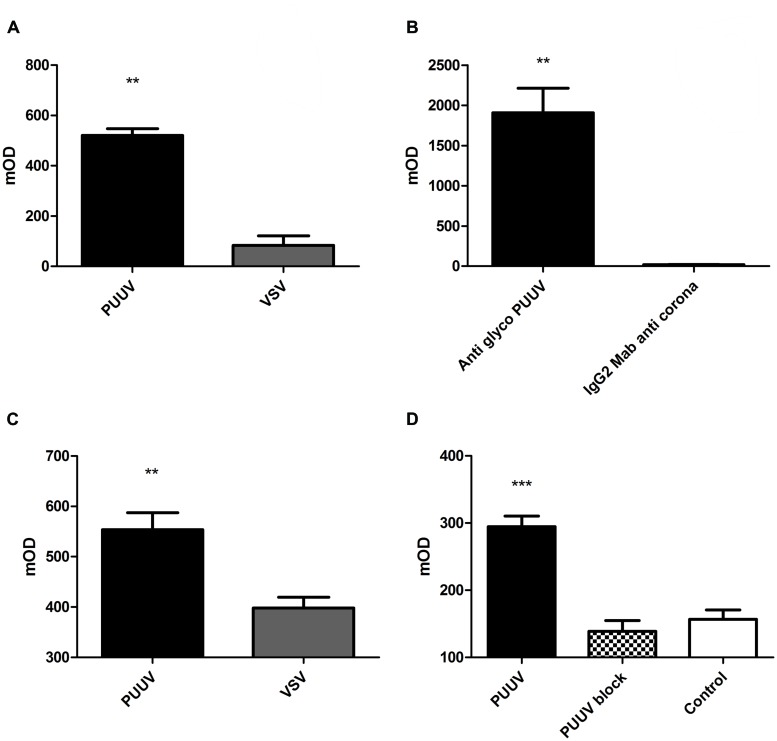
**Puumala in virus and platelets bind to each other.** In a pull down assay platelets adhere better to PUUV virus particles compared to vesicular stomatitis virus (VSV; **A**; Mann–Whitney *U* *p* 0.0022). When virus was captured with a PUUV glycoprotein antibody **(B)**, platelets were able to bind to the captured virus, in contrast to wells coated with an IgG2 control antibody (anti-coronavirus glycoprotein; Mann–Whitney *U* *p* 0.0022), resulting in no capture of PUUV during the incubation process, controlling for potential other factors present in the virus stock medium. When platelets were bound to plates coated with an anti-CD41a antibody **(C)**, the PUUV particles were able to bind to platelets based on the significant increase in PUUV detection OD compared to wells incubated with VSV particles, thus no PUUV present (Mann–Whitney *U* *p* 0.0043). The binding between PUUV and platelets could be blocked by the addition of a blocking step with human anti-PUUV serum **(D)** which show a decreased CD41 expression when compared to the PUUV coated wells incubated with a PUUV negative control serum. In all experiments no difference in OD was measured when an isotype control antibody was used. Data are representative of three independent experiments.**p < 0.01 ***p ≤ 0.001

Next we looked whether the platelets could bind directly to PUUV particles. For this purpose we performed an in-house developed platelet pull down-assay using quiescent platelets. To this end, several experiments were conducted to demonstrate specificity of this binding. **Figure 3** shows results of binding of platelets to virus-coated ELISA plates (**Figure 3A**). More platelets (Mann–Whitney *U*; *p* = 0.0022) adhered to plates directly coated with PUUV compared to plates coated with a virus control (VSV), which was cultured under the same conditions as PUUV.

Subsequently, to control if the binding of platelets was directly to the PUUV particles and not due to another factor present in the VeroE6 supernatant we made use of a sandwich ELISA principle. PUUV was incubated on ELISA plates with wells coated with a monoclonal IgG2 specific for the glycoprotein of PUUV or with a IgG2 control antibody. By this approach significantly more platelets bound to the wells where PUUV was captured compared to wells with no PUUV capture (Mann–Whitney *U*; *p* = 0.0022; **Figure 3B**).

To confirm direct binding between platelets and PUUV, platelets were captured to anti-CD41 coated ELISA plates and incubated with virus followed by detection with a PUUV specific antibody. To control for binding between PUUV detection antibody and captured platelets, control wells were incubated with VSV. PUUV detection was significantly higher in the wells incubated with PUUV compared to VSV (Mann–Whitney *U*; *p* = 0.0043; **Figure 3C**). These experiments collectively suggest that platelets can specifically bind to PUUV.

Finally, we show in **Figure 3D** that the binding of platelets to PUUV particles could be blocked by addition of a blocking step with human serum from a recovered PUUV case. When wells coated with PUUV were incubated with human serum with proven PUUV neutralizing IgG antibodies significantly less platelets adhered to the wells compared to wells incubated with a PUUV negative human control serum (**Figure 3D**; *p* < 0.001). The anti-CD41 expression in the wells with a blocking step was comparable to that of the negative control, which consisted out of plates coated with 5 days old vero E6 virus free medium. Binding of the neutralizing IgG antibodies was confirmed by incubation with a goat-anti human HRP labeled conjugate and subsequent TMB reaction.

### Increased Thrombin Generation and Tissue Factor Expression after PUUV Infection of HUVEC

**FIGURE 4:**
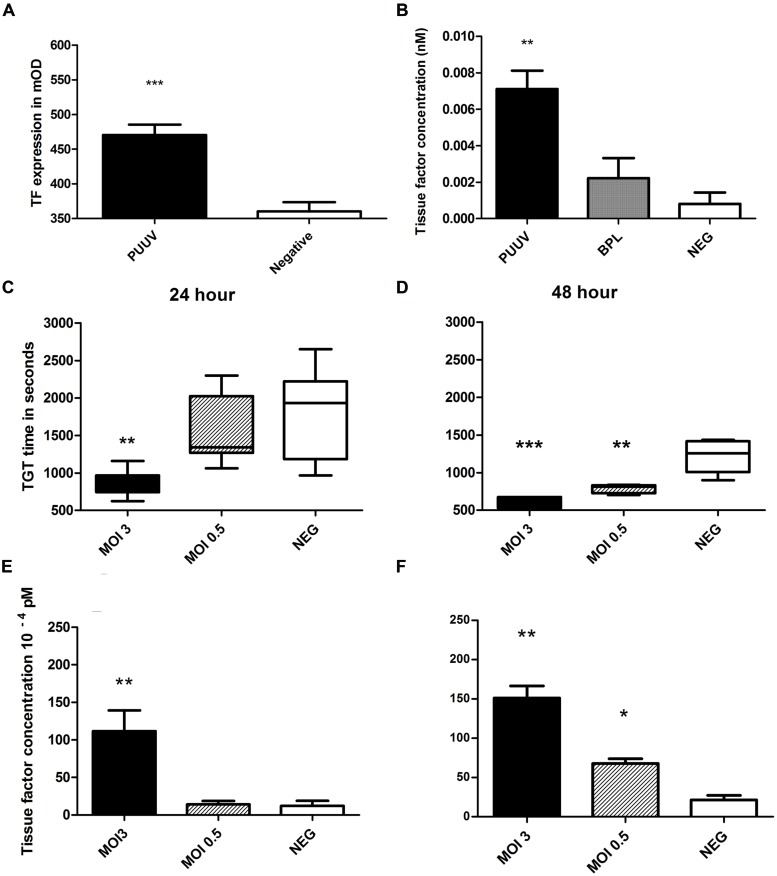
**Puumala virus infection of HUVEC induces tissue factor expression resulting in enhanced thrombin generation.** MOI 3 infection resulted in increased TF expression (**A**, Mann–Whitney *U* *p* = 0.0002) on HUVEC surface after 48 h **(A)** and in the cell lysate **(B)**. Thrombin generation time (TGT) was significantly decreased, indicating more thrombin formation, for cells infected with PUUV (MOI 3) at 24 **(C)** and 48 **(D)** hours post infection (one way ANOVA; 24 h *p* < 0.01 and 48 h *p* < 0.001). Results are shown with whiskers from minimum to maximum. MOI 0.5 infection resulted in shortened TGT 48 h post infection (one way ANOVA *p* < 0.001). Mean tissue factor (TF) concentration, calculated from TGT standard curve, increased more than ninefold after MOI 3 infection (one way ANOVA 24 and 48 h *p* < 0.01). MOI 0.5 infection increased TF concentration threefold after 48 h (one way ANOVA *p* < 0.05; **E, F**; bars represent SE of the mean). Data are representative of three independent experiments. ^∗^*p* < 0.05, ^∗∗^*p* < 0.01, ^∗∗∗^*p* ≤ 0.001.

To test the hypothesis whether increased thrombin generation observed in acute PUUV patients is the result of increased TF expression on endothelial cells we incubated HUVEC, infected with PUUV at a MOI of 3 or with a virus free 5 days old Vero E6 medium (control) with a polyclonal anti-TF antibody. By this approach we showed that TF expression was significantly increased with an almost twofold increase in OD value 48 h post infection (**Figure 4A**, Mann–Whitney *U*; *p* = 0.0047). Cells infected with PUUV also showed an increased TF concentration when the cell lysates of PUUV infected wells were compared to the lysates of control wells (**Figure 4B**; both mock and BPL). Subsequently we wanted to prove that the increase in TF expression on the endothelial cell surface was of biological significance and would led to increased thrombin generation. Thrombin generation was quantified directly on infected endothelial cells by incubating normal plasma on cells and initiating coagulation by the addition of calcium ions. Infected cells induced plasma clotting faster due to increased thrombin generation (**Figures 4C,D**). Using a calibration curve with purified TF in the absence of endothelial cells, we quantified HUVEC TF production after virus infection and after incubation with a virus free medium control. TF concentration showed a statistically significant increase for MOI 3 at 24 h compared to the negative control and the HUVEC infected with MOI 0.5 (one way ANOVA; *p* < 0.01) and at 48 h post infection compared to the negative control (one way ANOVA; *p* < 0.001). The MOI 0.5 infection led to higher levels of TF on the HUVEC surface only after 48 h post infection (one way ANOVA; *p* < 0.05) when compared to mock.

### Increased Levels of PAI-1 and PAI-1-Vitronectin Complexes in HUVEC Supernatant after Infection with PUUV

**FIGURE 5:**
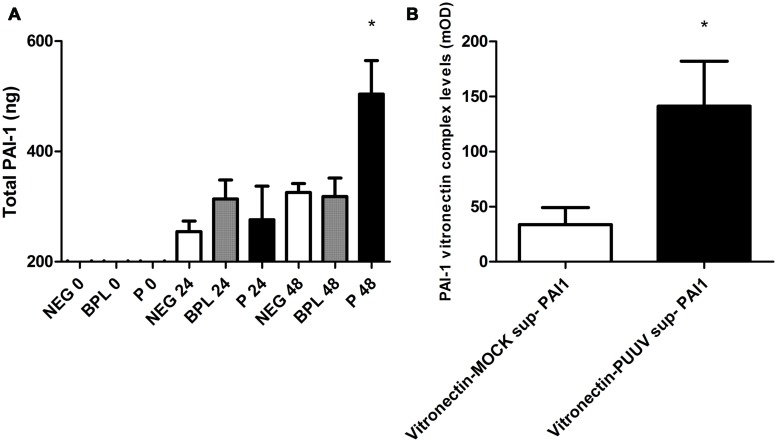
**Plasminogen activator inhibitor type 1 production, surface expression of ανβ3-integrin and PAI-1 vitronectin complex formation in HUVEC infected with PUUV.** Total plasminogen activator inhibitor type-1 (PAI-1) concentration was significantly increased on time points 48 post PUUV infection (black; one way ANOVA, *p* < 0.05; **A**) compared to BPL inactivated control (gray) or negative medium control (white). Panel B shows the PAI-1 vitronectin complex levels in HUVEC infected with PUUV (black) or non-infected controls (white). After 48 h of infection PAI-1 vitronectin levels were increased compared to the mock infection (Mann–Whitney *U* *p* = 0.03; **B**). Data are representative of three independent experiments. ^∗^*p* < 0.05.

Important proteins in the regulation of fibrinolysis show close interactions with the pathogenic hantavirus receptor ανβ3 integrin. For instance vitronectin, a stabilizer of PAI-1 activity in plasma, is largely regulated by this receptor (Mackow and Gavrilovskaya, 2009; Florova et al., 2013). To study potential changes in regulators of fibrinolysis we first measured PAI-1 levels in the cell-free supernatant and supernatant of cell lysate from 24-well plates infected with PUUV or control infections. The total PAI-1 antigen (the combination of levels in the supernatant and cell lysate) was significantly increased 48 h post infection (Kruskal–Wallis; *p* < 0.05) with MOI 3 (**Figure 5A**). Subsequently, we tested if in our model PAI-1 would bind to vitronectin, since this binding is associated with increased/prolonged PAI-1 activity (Seiffert and Loskutoff, 1991), and if this interaction is altered during infection. ELISA plates coated with a monoclonal antibody against vitronectin, incubated with supernatant from our experiments (pooled, control or from MOI 3 infected wells) followed by incubation with PAI-1 antibody suggested formation of PAI-1 vitronectin complexes due to an increase in OD compared to incubation with PBS (mean expression in medium 490 mOD (±100) vs. 370 mOD (±70) p = 0.02). If supernatants were tested separately (PUUV vs. mock) levels of PAI-1 vitronectin complexes were increased after PUUV infection (Mann–Whitney *U*; *p* = 0.03; **Figure 5B**).

## Discussion

The present study addresses platelet binding to PUUV infected cells and activation of secondary hemostasis after endothelial cell PUUV infection. With the lack of a valid and accessible animal model for old-world hantavirus infection, we remain dependent on *ex vivo* cell culture models to address questions regarding virulence and pathogenesis (Vaheri et al., 2013b). Taking into account the recently found association of PUUV infection with cardiovascular disease (Connolly-Andersen et al., 2014) and hemorrhagic complications that may occur during infection, the interaction between PUUV and the coagulation system especially warrants further attention. Since PUUV tends to rapidly lose virulence upon *in vitro* cell passages the use of low passaged isolates is of vital importance (Nemirov et al., 2003). Therefore, we have put a lot of emphasis on obtaining low passage PUUV isolates and optimisation of the hemostatic assays under the right biosafety regulations using primary cell cultures.

Based on hemostatic changes seen in several clinical studies, most from Northern Europe, we decided to study specific parts of the coagulation system *in vitro*. We started by studying the effects of PUUV infection on formation of a platelet plug, the major event in primary hemostasis. Binding of platelets by PUUV infected cells could explain thrombocytopenia in acute PUUV patients, since it would result in wasting or loss of platelets adhered to these cells (Gavrilovskaya et al., 2010; Laine et al., 2011)*.* Especially if we make notice of the ability of hantaviruses to infect megakaryocytes and thereby lead to a decreased production of platelets, in addition to the loss of platelets adhered to infected cells (Liang et al., 2004; Lutteke et al., 2010). In our model it seems that PUUV infection increases binding of platelets to the surface of HUVEC compared to control cells (**Figure 2**). Here we assumed increased CD41a expression observed in the first experiments was the result of an increased number of platelets on the HUVEC. Theoretically, increased CD41a detection could also be due to an increased expression of CD41a on platelets, after 30 min incubation with infected HUVEC, rather than an actual increase in platelet numbers. While we cannot rule this out based on our experiments, we blocked extrinsic platelet activation by prostaglandin treatment making platelet activation less likely. Furthermore, in line with studies performed with more pathogenic hantaviruses (Gavrilovskaya et al., 2010), we tested specific binding of platelets to PUUV particles. Judged from results from the platelet pull down experiments (**Figure 3**) this seemed to be a specific binding between virus and platelets which could be reversed by the addition of PUUV neutralizing antibodies. In these experiments we controlled for aspecific binding of antibodies (isotype control experiments), factors present in the virus culture medium (5 days old VeroE6 medium as a control) and binding of platelet detection antibody directly to PUUV.

We expected the increased platelet binding to co-occur with increased VWF production, as a general inflammatory response during infection. However, the observation that VWF concentration does not change during PUUV infection, further suggested a VWF-independent mechanism for platelet binding in HFRS. Results from earlier studies showed an increased VWF concentration in hospitalized PUUV patients (Laine et al., 2011). One should keep in mind that overall plasma VWF level in any patient represents the state of the total endothelial cell layer and not only that of infected cells, as is the case in our model. Furthermore we are studying the acute response of endothelial cells in the first 48 h after infection, a time point at which PUUV patients are generally not considered to be hospitalized and tested. The increase of VWF in all three conditions (control, BPL and PUUV) over time in our HUVEC model could be the result of an increased number of cells or a sign of *in vitro* stress and activation of the endothelial cells. Since it seems highly unlikely the cells still multiply after the formation of a full monolayer, which is present at the time of infection, we believe that also non-infected cells show a certain level of activation when in culture.

Gavrilovskaya et al. (2010) were the first to study the interaction of hantaviruses (Andes and Hantaan) with platelets, and concluded that there was a specific binding of Hantaan and Andes virus particle particles to ανβ3 integrins present on both endothelial cells and platelets. Interestingly, our experiment showed a trend to increased number of platelets bound to the BPL-inactivated virus treated cultures, suggesting active replication was unnecessary and inactivated virus, bound to the cell surface, might also bind to integrins present on platelets. However, this hypothesis is merely based on a statistical trend observed in wells with the cells incubated with BPL inactivated virus. For the interpretation of our data one should take in mind that we made use of a MOI 3 BPL at *t* = 0 h and that the BPL inactivated virus will not replicate. Therefore at timepoint *t* = 24 and *t* = 48 the BPL control will most likely be comparable to the MOI 0.5 infection. It could very well be the case that when increasing the MOI for the BPL infection a more comparable result to the MOI 3 infection would be observed.

Puumala virus infections of HUVEC directly increased the expression of TF on the cell surface and in the cell lysate compared to controls. This resulted in drastic activation of secondary hemostasis in our cell model during PUUV infection. Data from a direct clotting assay on the cell monolayer gives interesting insights in the potential mechanism behind increased thrombin generation seen in acute PUUV patients (Laine et al., 2010, 2014). A clear pro-coagulant state, the result of an increased expression of TF on the surface of PUUV infected cells, resulted in enhanced thrombin generation. Increased thrombin generation (decreased thrombin time, overall increase in prothrombin fragments 1 + 2, antithrombin and protein C) that Laine et al. (2010, 2014) observed in acute PUUV patients could very well be the result of direct infection of endothelial cells and concomitant increased production of TF. Whether increase in TF is a general defense response or if the virus actually benefits from TF, as is seen in certain herpesvirus infections (Pryzdial et al., 2014), remains unknown. However, excess of TF production during infection could lead to increased clotting and eventually consumptive coagulopathy or even DIC, a severe condition that is only seen in a small percentage of PUUV patients (Laine et al., 2010), but which could be one of the factors contributing to the hemorrhagic complications seen in HFRS. Especially since increased TF expression has been proven to play an important role in the pathogenesis of other viral hemorrhagic fevers like Marburg and Ebola (Geisbert et al., 2003a,b).

Since alterations in PAI-1 levels are related to renal disturbances comparable to that seen in hantavirus disease (Gong et al., 2007; Malgorzewicz et al., 2013) and functional polymorphisms in PAI-1 were related to more severe disease in acute PUUV patients (Laine et al., 2012), we also studied PAI-1 and regulators of PAI-1 activity. Infection with PUUV increases PAI-1 production, which would *in vivo* lead to decreased fibrinolysis. The ανβ3 integrin receptor plays an important role in PAI-1/vitronectin complex formation (Zhou et al., 2003). Increased ανβ3 expression during PUUV infection combined with competitive binding of hantavirus with vitronectin for ανβ3 could hypothetically lead to further alterations in PAI-1 half-life and stability. The increased level of vitronectin-PAI-1 complexes in the supernatant of PUUV infected cells further strengthens this hypothesis. Considering that an increase in PAI-1 and vitronectin could result in renal impairment, and even cause a nephritis-like response, pledges for further evaluation of interaction between ανβ3 integrin, PAI-1, vitronectin and hantaviruses.

## Author Contributions

MG was the primary investigator in this study, he performed (most) of the experiments and data analyses. JM, E and BM supervised the experiments, raw data analysis and hypothesis formation. FA and CW contributed with the design, optimisation and maintenance of the primary HUVEC culture. JR (viral kinetics) and KB (cell thrombin generation test) each contributed with the design and implementation of tests for this study. HH and AV made substantial contribution by assisting with the cultivation of low passage PUUV isolates. MG, JM, FA, JR, CW, KB, HH, AV, AO, EG and BM all contributed in the planning of the manuscript, data analysis and interpretation, and critical review and approval of the manuscript.

## Conflict of Interest Statement

Albert D. M. E. Osterhaus is a consultant to Viroclinics Biosciences BV, a spin out of Erasmus MC. The authors declare that the research was conducted in the absence of any commercial or financial relationships that could be construed as a potential conflict of interest.
